# Supplementary material for: LRG1 promotes proliferation and inhibits apoptosis in colorectal cancer cells via RUNX1 activation
Source: PLoS One. 2017 Apr 4;12(4):e0175122. doi: 10.1371/journal.pone.0175122 (PMC5380360; doi:10.1371/journal.pone.0175122)
Supplement: S1 Manuscript — The language of the manuscript was polished by American Journal Experts. (DOCX) [file pone.0175122.s002.docx]

LRG1 promotes proliferation and inhibits apoptosis in colorectal cancer cells via RUNX1 activation

Ying Zhou^1¶^, Qingwei Zhang^1¶^, Jingyuan Fang^1^, Zhizheng Ge^1^ and Xiaobo Li^1*^

^1^Division of Gastroenterology and Hepatology, Key Laboratory of Gastroenterology and Hepatology, Ministry of Health, Renji Hospital, School of Medicine, Shanghai Jiao Tong University; Shanghai Institute of Digestive Disease;145 Middle Shandong Road, Shanghai 200001, China.

* Corresponding author:

E-mail: [lxb_1969@163.com](mailto:lxb_1969@163.com) (XBL)

^¶^Ying Zhou and Qingwei Zhang are both first authors. Ying Zhou and Qingwei Zhang contributed equally to this work.

**Authors’ contributions：**YZ played roles in **Conceptualization, Data Curation, Formal Analysis, Investigation, Methodology, Resources, Validation, Visualization, and Writing – Original Draft Preparation and Writing – Review & Editing.** QWZ played roles in **Conceptualization, Data Curation, Formal Analysis, Investigation, Resources, Validation and Original Draft Preparation and Writing.** JYF and ZZG contributed to the **Funding Acquisition, Methodology, Visualization** and **Project Administration.** XBL contributed to the **Conceptualization, Data Curation,** **Investigation, Methodology, Resources, Supervision, Visualization and Writing – Review & Editing.**

**Abstract**

Leucine-rich-alpha-2-glycoprotein 1 (LRG1) has been shown to be involved in various human malignancies. Whether it plays a role in colorectal cancer (CRC) development remains unclear. Here, we investigated whether and through what mechanism LRG1 functions in human CRC cells. The plasma level of LRG1 was significantly increased in CRC patients, but it was remarkably decreased in patients with resected colorectal cancers. The knockdown of LRG1 significantly inhibited cell proliferation, induced cell cycle arrest at the G0/G1 phase, and promoted apoptosis in SW480 and HCT116 cells in vitro. In addition, LRG1 silencing led to the downregulation of the levels of key cell cycle factors, such as cyclin D1, B, and E and anti-apoptotic B-cell lymphoma-2(Bcl-2). However, it up-regulated the expression of pro-apoptotic Bax and cleaved caspase-3. These conclusions were based on both real-time PCR and Western blot analysis. Furthermore, RUNX1 could be induced by LRG1 in a concentration-dependent manner, while the knockdown of RUNX1 blocked the promotion of the proliferation and inhibition of apoptosis induced by LRG1. Collectively, these findings indicate that LRG1 plays a crucial role in the proliferation and apoptosis of CRC by regulating RUNX1 expression. Thus, it may be a potential biomarker and therapeutic target for CRC.

**Keywords:** LRG1, RUNX1, proliferation, apoptosis, colorectal cancer.

**Abbreviations**

LRG1: leucine-rich-alpha-2-glycoprotein 1; CRC: colorectal cancer; ELISA: enzyme-linked immunosorbent assay; RUNX: runt-related transcription factors; LGN: low-grade neoplasia; ESD: endoscopic submucosal dissection.

**Introduction**

Colorectal cancer (CRC) is the third most common cancer and the fourth leading cause of cancer-related deaths worldwide^[^[^1^](#_ENREF_1)^]^. In view of its high morbidity and mortality, thorough screening methods and treatments are urgently needed. Currently, colonoscopies as well as the plasma levels of CEA are extensively used as screening methods throughout the world^[^[^2^](#_ENREF_2)^,^ [^3^](#_ENREF_3)^]^. However, a number of patients do not undergo colonoscopy at recommended intervals. On the other hand, CEA lacks the specificity and sensitivity as a diagnostic marker for CRC^[^[^4^](#_ENREF_4)^]^, and the pathogenesis of CRC has not yet been fully elucidated. Therefore, it is important to determine the molecular mechanism for CRC development to recognize novel biomarkers and establish therapeutic targets for CRC.

Leucine-rich-alpha-2-glycoprotein1 (LRG1), a membrane-associated leucine-rich repeat (LRR) family member, was isolated from human serum by Haupt and Baudner in 1977^[^[^5^](#_ENREF_5)^]^. LRG1 is induced by proinflammatory cytokines^[^[^6^](#_ENREF_6)^]^. The expression of LRG1 is overexpressed in paediatric appendicitis, ulcerative colitis, inflammation, the immune response and neovascularization^[^[^7-10^](#_ENREF_7)^]^. Moreover, LRG1 has been shown to be up-regulated in several types of carcinomas, such as hepatocellular carcinoma, gastric cancer, pancreatic cancer, leukaemia, ovarian cancer, bladder cancer and non-small cell lung cancer^[^[^11-17^](#_ENREF_11)^]^. However, the biological function of LRG1 in the tumourigenesis and progression of colorectal cancer is not yet clear.

To investigate the downstream signalling of LRG1, we performed a gene microarray and found that the runt-related transcription factors (RUNX) were affected by the depletion of LRG1 in CRC cells. The RUNX family contains three members, RUNX1, RUNX2 and RUNX3. They function as either transcriptional repressors or activators, depending on the different genes and cell types; additionally, they have tissue-specific properties^[^[^18^](#_ENREF_18)^]^. RUNX1 has been shown to play a role in haematopoiesis and haematopoietic function^[^[^19^](#_ENREF_19)^]^. RUNX2 has been reported to be a key regulator required for osteogenic differentiation^[^[^20^](#_ENREF_20)^]^, while RUNX3 has been shown to be related to gastrointestinal tract development^[^[^21^](#_ENREF_21)^]^. All three RUNX genes are associated with Smads and the TGF-β signalling pathway^[^[^22-24^](#_ENREF_22)^]^. However, little is known about the relationship between LRG1 and RUNX genes in CRC.

In the present study, we explored the plasma levels of LRG1 in CRC patients and investigated the role of LRG1 in CRC cell proliferation and apoptosis. Additionally, this study aimed to determine which RUNX genes mediated by LRG1 act as downstream effectors in the proliferation and apoptosis progress of human CRC cells.

**Materials and methods**

**Cell culture and LRG1 treatment**

The human colorectal carcinoma cell lines SW480 and HCT116 were obtained from the China Center for Type Culture Collection (Beijing, China). Both cell lines were cultured in RPMI 1640 medium supplemented with 10 % foetal bovine serum (FBS, Invitrogen, California, USA) in a humidified 5.0 % CO2 atmosphere at 37 °C. Recombinant LRG1 was purchased from Biorbyt and added to the culture medium at concentrations of 50–1,000 ng/ml for the indicated time before harvesting.

**Small interfering RNA silencing**

Small interfering RNAs (siRNAs) were purchased from GenePharma (China) in addition to the oligos for LRG1 (#1: sense, 5’ -GCAAUUAGAACGGCUACAUT T-3’ and antisense, 5’ -AUGUAGCCGUUCUAAUUGCTT-3’; #2: sense, 5′-CCUCUAAGCUCCAAGAAUUTT-3’ and antisense, 5′-AAUUCUUGGAGCUUAGAGGTT-3′), RUNX-1 (sense, 5’ -CCAGGUUGCAAGAUUUAAUTT-3’ and antisense, 5’ -AUUAAAUCUUGCAACCUGGTT-3’), and a non-targeting control siRNA. Transfection of siRNAs was performed using Lipofectamine 2000 (Invitrogen, USA) according to the manufacturer’s protocol. The culture medium was exchanged 6 h after transfection, and the cells were harvested 24 h or 48 h later.

**RNA extraction and quantitative real-time PCR**

The total RNA was extracted using TRIzol reagent (Invitrogen, USA), and cDNA was synthesized using the PrimeScriptTM RT Reagent Kit (Perfect Real Time, TaKaRa, Japan). Quantitative real-time PCR was performed in a total volume of 10 µl containing SYBR Green (SYBR® Premix Ex TaqTM II, TaKaRa, Japan) on an Applied Biosystems 7900 quantitative PCR system. After normalization to the β-actin gene, the relative expression of each target gene was determined according to the 2−ΔΔCt method. The data are presented as the means ± standard deviation (SD) from three independent experiments.

**Protein extraction and Western blot analysis**

Whole cellular proteins were isolated from fresh cells with lysis buffer containing RAPI PMSF. Protein concentrations in the supernatants were measured using a BCA protein assay. Equal amounts of total protein were loaded onto a 10% SDS-PAGE gel, transferred onto polyvinylidene difluoride membranes, and blocked with 5% fat-free milk for 2 h at room temperature. The membranes were incubated overnight at 4°C with a primary antibody, and this step was followed by treatment with horseradish peroxidase-conjugated secondary antibodies for 1 h at room temperature. Immunodetection was performed using the Super Signal West Femto Maximum Sensitivity Substrate (Thermo Fisher, Massachusetts, USA). The antibody for LRG1 was purchased from Sigma (HPA001888, rabbit polyclonal), and the other antibodies were all purchased from Cell Signaling Technology, Inc. (USA). The intensity of the target protein bands was determined using the ImageJ software and normalized to that of β-actin.

**Evaluation of cell proliferation**

To evaluate cell proliferation, SW480 and HCT116 cells were seeded in 96-well microplates at a density of 3×10^3 cells/well. At the indicated time points (0, 24, 48, 72, or 96 h), 10 μL of CCK-8 solution (DOJINDO, Japan) was added to 100 μL of culture medium. After 2 h of incubation at 37°C, the absorbance of the culture medium was measured at 450 nm (A450) using a scanning microplate spectrophotometer (Multiscan GO, Thermo Fisher Scientific, Waltham, MA, USA).

**Evaluation of cell cycle**

Forty-eight hours after transfection, the cells were collected, washed with ice-cold phosphate buffer solution (PBS), and fixed in 75% ethanol at -20°C overnight. Prior to staining, the cells were washed with PBS. The cells were treated with 10 μL RNase A (10 mg/mL, TaKaRa, Japan) for 30 min at 37 °C and then stained with 50 μL of a propidium iodide (PI) (250 μg/mL) solution. In total, 30,000–50,000 cells were analysed on a FACScan flow cytometer (Becton–Dickinson LSRFortessa, San Jose, CA, USA) and analysed with FlowJo V10 software.

**Evaluation of cell apoptosis**

Cell apoptosis was probed using the FITC Annexin V/PI Apoptosis Detection Kit (BD Pharmingen, USA) according to the manufacturer’s instructions. In brief, cells were collected, washed with Binding Buffer at 4°C, and resuspended in 200 μL of Binding Buffer. A total of 1 × 10^6 cells were stained with 2.5 μL of Annexin V-FITC and 5 μL of PI. Cells were then incubated with the staining solution at room temperature in the dark. In total, 30,000–50,000 cells were analysed on a FACScan flow cytometer (Becton–Dickinson LSRFortessa, San Jose, CA, USA) and analysed with FlowJo V10 software.

**Clinical specimens and enzyme-linked immunosorbent assay**

Thirty patients with colorectal cancer, 30 patients with adenoma or low-grade neoplasia (LGN), 30 CRC patients who underwent endoscopic submucosal dissection (ESD) or surgery and 30 matched normal patients were enrolled at Shanghai Renji Hospital. All cases were individually matched based on age (within 3 years) and gender. Plasma samples were obtained from the above patients. The study was approved by the Ethics Committee of Renji Hospital with written informed consent from all patients included in this study. Human LRG1 was quantified using an enzyme-linked immunosorbent assay (ELISA) kit (R&D Systems Europe, UK) according to the manufacturer’s instructions.

**Statistical analysis**

The data and figures were processed using the GraphPad Prism 5.0 software (San Diego, CA, USA). Quantitative data were expressed as the means ± SEM, and comparisons between two groups were performed using Student’s t test or a paired t test. P-values <0.05 were considered statistically significant.

**Results**

**Increased plasma level of LRG1 was related to human CRCs**

To investigate the correlation between the plasma level of LRG1 and human CRCs, we collected plasma samples from patients who underwent coloscopy examination and ESD or surgery at Renji Hospital. Fig. 1 shows that the plasma level of LRG1 was increased in adenomas (LGN) and CRCs, and the concentration of LRG1 was significantly increased in human CRCs compared with adenomas and controls. Furthermore, the plasma level of LRG1 was remarkably decreased in patients who had resected colorectal cancers, which was consistent with normal patients.

**LRG1 influenced CRC cell** **proliferation and cell cycle arrest**

The increased plasma level of LRG1 in CRCs suggested that LRG1 might play a role in its proliferation capability. To investigate the role of LRG1 in CRC cells, we knocked down LRG1 using the transfection of specific siRNA. Both the mRNA and protein levels of LRG1 were significantly down-regulated in SW480 and HCT116 cells (Fig. 2a). According to the performed CCK-8 assays, the knockdown of LRG1 efficiently reduced the proliferation rates of SW480 and HCT116 cells (Fig. 2b), implying that LRG1 silencing inhibited the growth of SW480 and HCT116 cells. Furthermore, the knockdown of LRG1 resulted in the accumulation of cells in the G0/G1 phase and decreased the number of cells in the S and G2/M phases, which was more significant in SW480 cells than HCT116 cells (Fig. 2c-d). Next, we investigated the expression of cell cycle markers following the knockdown of LRG1. Real-time RT-PCR and Western blot analysis showed that the expression levels of cyclin D1, B, and E in LRG1-knockdown cells were significantly decreased compared with NC cells (Fig. 2e–f). In summary, LRG1 silencing suppressed cell growth by inhibiting cell proliferation as well as inducing cell cycle arrest in SW480 and HCT116 cells.

**LRG1 influenced CRC** **cell apoptosis**

The cell apoptotic status was analysed using an FITC annexin V/PI double staining assay. As shown in Fig. 3a, b, the number of apoptotic cells was markedly increased in LRG1-silenced SW480 and HCT116 cells compared with NC cells (P<0.001). We further examined the mRNA and protein levels of the apoptotic suppressor B-cell lymphoma-2(Bcl-2) as well as its downstream apoptotic promoters cleaved caspase-3 and Bax by real-time PCR and Western blot analysis. The results showed that the knockdown of LRG1 significantly down-regulated the levels of Bcl-2, whereas it elevated the expression of Bax and cleaved caspase-3 in both SW480 and HCT116 cells (Fig. 3c-d). The above findings provide evidence for the role of LRG1 silencing in enhanced apoptosis in SW480 and HCT 116 cells.

**RUNX1 was essential for LRG1-mediated proliferation and** **apoptosis**

The RUNX family is believed to play an essential role in carcinogenesis and normal development. To investigate whether LRG1 could regulate the expression of RUNXs, we analysed the relative mRNA expression of the RUNX family in LRG1-silenced CRC cells. Real-time RT-PCR data showed that RUNX1 expression was significantly decreased in LRG1-silenced CRC cells, while the knockdown of LRG1 had no effect on the levels of RUNX2 and RUNX3 (Fig. 4a). To determine whether LRG1 could enhance RUNX1 expression, CRC cells were stimulated with rLRG1 for 24 h. The results showed that the mRNA expression of RUNX1 was elevated when treated with 100 ng/ml rLRG1 and reached a maximum level at 1,000 ng/ml rLRG1 (Fig. 4b). LRG1 stimulation also resulted in the promotion of the growth of SW480 and HCT116 cells (Fig. 4c). Moreover, the protein expression levels of RUNX1 as well as cyclin D1, B, E and Bcl-2 were also increased, while protein expression of Bax and cleaved caspase-3 were decreased in a concentration-dependent manner following stimulation with rLRG1 in SW480 cells (Fig. 4d). Furthermore, we investigated the role of RUNX1 in LRG1-induced proliferation and apoptosis. Transfection with siRNA successfully downregulated the mRNA and protein levels of RUNX1 in SW480 cells (Fig. 5a). As shown in Fig. 5b-f, silencing RUNX1 inhibited the enhanced proliferation and anti-apoptosis ability of CRC cells induced by rLRG1 and resulted in cell cycle arrest in SW480 cells. The knockdown of RUNX1 also reversed LRG1-induced expression of cell cycle- and apoptosis-associated proteins, increasing the expression of Bax and cleaved caspase-3 and decreasing the expression of cyclin D1, B, E and Bcl-2 (Fig. 5g). The above results strongly suggest that RUNX1 is involved in LRG1-induced CRC cell proliferation, cell cycle and apoptosis.

**Discussion**

It was reported that LRG1 expression was significantly higher in CRC tissues compared with normal tissues^[^[^25^](#_ENREF_25)^]^. In this study, we demonstrated that the plasma level of LRG1 was increased in adenomas (LGN) and CRCs, and the concentration of LRG1 was remarkably increased in human CRCs compared with adenomas and controls, which was consistent with data found in the literature^[^[^6^](#_ENREF_6)^,^ [^26^](#_ENREF_26)^]^. Moreover, we found that the plasma level of LRG1 was significantly decreased in patients who have undergone CRC resections. This result indicates that LRG1 might be a novel biomarker for predicting and following-up postoperative CRCs. Additionally, the high plasma level of LRG1 in CRCs suggests that LRG1 expression might affect the growth and apoptosis of colorectal carcinoma cells.

Although LRG1 was shown to play a role in a variety of benign and malignant diseases, it has been reported that LRG1 is a tumour suppressor in endometrial and hepatocellular carcinoma^[^[^27^](#_ENREF_27)^,^ [^28^](#_ENREF_28)^]^. However, the molecular mechanism of LRG1 has not been fully elucidated thus far. Recent studies showed that LRG1 promotes pathogenic neovascularization, cell adhesion, cell migration, and cell invasion and is involved in the epithelial-to-mesenchymal transition (EMT) process^[^[^25^](#_ENREF_25)^,^ [^29^](#_ENREF_29)^,^ [^30^](#_ENREF_30)^]^. Our study demonstrated that the silencing of LRG1 expression significantly inhibited the growth of colorectal carcinoma cells; meanwhile, stimulation with recombinant LRG1 enhanced cell proliferation.

Proliferation and the cell cycle play an important role in the carcinogenesis of tumours. The cell cycle of eukaryotic cells is the essential process of cell proliferation^[^[^31^](#_ENREF_31)^]^. In our study, the knockdown of LRG1 by siRNA resulted in colorectal cancer cell cycle arrest with the accumulation of cells in the G0/G1 phase and reduced the cell numbers in the S and G2/M phases, while stimulation with recombinant LRG1 had an inverse effect. Moreover, we demonstrated that silencing LRG1 expression led to the significant downregulation of cell cycle genes, including cyclin D1, B, and E, which have been reported to play crucial roles in the transition of the G1/S phase during cell proliferation and the cell cycle^[^[^32^](#_ENREF_32)^]^ and the over-expression of these genes lead to a shortened G1 phase and accelerated tumour formation ^[^[^33^](#_ENREF_33)^]^. The results imply that LRG1 modulates the cell cycle and cell proliferation of colorectal cancer by regulating the expression of cyclin D1, B, and E.

The imbalance of cell apoptosis also leads to tumourigenesis. The stable knockdown of LRG1 has been reported to promote the apoptosis of glioblastoma cells^[^[^34^](#_ENREF_34)^]^. However, the role of LRG1 in colorectal cancer cell apoptosis has not been addressed. In this study, the knockdown of LRG1 by siRNA significantly promoted the apoptosis of both SW480 cells and HCT116 cells, while stimulation with recombinant LRG1 remarkably inhibited SW480 cell apoptosis. Furthermore, LRG1 silencing significantly down-regulated both the mRNA and protein expression of the anti-apoptotic protein Bcl-2 but up-regulated both the mRNA and protein expression of pro-apoptotic protein Bax and cleaved caspase-3. The results presented above suggest that LRG1 may be involved in the apoptosis of colorectal cancer cells through these classic apoptotic proteins.

The RUNXs are believed to play an essential role in carcinogenesis in addition to their role in normal development^[^[^35^](#_ENREF_35)^]^. Among the three RUNXs, gene polymorphisms and hypermethylation in RUNX3 have been reported to be related to colorectal cancer^[^[^36^](#_ENREF_36)^,^ [^37^](#_ENREF_37)^]^. However, as all three RUNX genes are involved in signalling cascades mediated by bone morphogenetic protein (BMP) and TGF-β, they all have the potential to participate in CRC aetiology^[^[^38^](#_ENREF_38)^,^ [^39^](#_ENREF_39)^]^. A recent study showed that genetic variation in three RUNXs was associated with high risk for colon and rectal cancers^[^[^40^](#_ENREF_40)^]^. Our result revealed that LRG1 silencing significantly down-regulated the expression of RUNX1 rather than RUNX2 and RUNX3. Additionally, the mRNA and protein expression level of RUNX1 was increased in a concentration-dependent manner following stimulation with rLRG1 in CRC cells. Additionally, our results indicated that RUNX1 is involved in LRG1-induced proliferation and apoptosis since the knockdown of RUNX1 blocked the promotion of the proliferation and inhibition of apoptosis induced by LRG1. Moreover, RUNX1 knockdown reversed the LRG1-induced up-regulation of cyclin D1, B, E and Bcl-2 and the induced down-regulation of Bax and cleaved caspase-3, which act as key factors in proliferation and apoptosis. These data suggest that LRG1 may target the RUNX1 pathway to induce CRC cell proliferation and apoptosis. As both LRG1and RUNX1 are involved in the TGF-β signalling pathway^[^[^10^](#_ENREF_10)^,^ [^41^](#_ENREF_41)^]^, it is not clear whether LRG1 activates RUNX1 transcription directly or via the TGF-β pathway.

In summary, our results provide evidence for the use of LRG1 as a novel plasma biomarker for the prediction of CRCs as well as postoperative following-up for CRCs. We demonstrated that LRG1 promotes cell proliferation, while inhibiting apoptosis, in CRC cells through the promotion of RUNX1 expression. Thus, LRG1 may act as a potential biomarker and a new therapeutic target for colorectal cancer.

**Acknowledgements**

This study was supported by the Innovation Program of Shanghai Municipal Education

Commission (No.16ZR1420400).

**Figure legends**

**Fig. 1 The plasma level of LRG1 was increased in human CRCs.**

Plasma samples from 30 CRC patients, 30 adenoma patients, 30 CRC patients after surgery and 30 normal patients were obtained, and the secretion of LRG1 was quantified by an ELISA.

**Fig. 2** **The knockdown of LRG1 inhibited** **CRC cell proliferation and induced cell cycle arrest. (a)** RT-PCR and Western blot analyses showed that LRG1 was effectively down-regulated by siRNA transfection. (**b)** A CCK-8 assay was performed to evaluate cell proliferation in vitro. (**c-d)** SW480 and HCT116 cells were fixed, stained with PI, and subjected to flow cytometry analysis for cell cycle. (**e–f)** The expression levels of cyclin D1, B, and E in SW480 and HCT116 cells transfected with LRG1 siRNA or control siRNA were determined by RT-PCR and Western blot analysis. This figure shows representative images of repeated experiments, and the data are presented as the mean±standard deviation. Compared with NC, *P<0.05, **P<0.01, and ***P<0.001.

**Fig. 3 The knockdown of LRG1 promoted CRC cell apoptosis.**

**(a-****b)** SW480 and HCT116 cells were double stained with an FITC-conjugated anti-Annexin V antibody and PI and then subjected to flow cytometry for cell apoptosis analysis. The cells in the upper right and lower right quadrants were considered late and early apoptotic cells, respectively. **(c-****d)** The expression levels of Bcl-2, Bax, and cleaved caspase-3 in SW480 and HCT116 cells transfected with LRG1 siRNA or control siRNA were determined by RT-PCR and Western blot analysis. This figure shows representative images of repeated experiments, and the data are presented as the mean±standard deviation. Compared with NC, **P<0.01 and ***P<0.001.

**Fig. 4 LRG1 promoted the expression of RUNX1 and CRC cell proliferation.**

**(a)** SW480 and HCT116 cells were transfection with LRG1 siRNA, and the expression of the tested RUNXs was quantified by RT-PCR. (**b)** Cells were stimulated with rLRG1 (0–1,000 ng/ml) for 24 h, and the RUNX1 expression was determined by RT-PCR. (**c)** Cells were stimulated with rLRG1 (0–1000 ng/ml), and a CCK-8 assay was performed to evaluate cell proliferation. (**d)** The protein levels of cyclin D1, B, E, Bcl-2, Bax, and cleaved caspase-3 in response to LRG1 treatment in SW480 cells were analysed by Western blot. This figure shows representative images of repeated experiments, and the data are presented as the mean±standard deviation. Compared with NC, *P<0.05, **P<0.01, and ***P<0.001.

**Fig. 5 The role of RUNX1 in LRG1-induced cell proliferation and anti-apoptosis.**

(**a)** RUNX1 expression was knocked down in siRNA-transfected SW480 cells at both the mRNA and protein levels. **(b)** SW480 cells were transfected with RUNX1 or control siRNA with or without rLRG1 (500 ng/ml) stimulation, and a CCK-8 assay was performed to evaluate cell proliferation. **(c-d)** SW480 cells were transfected with HIF-1α or control siRNA for 24 h and treated with or without rLRG1 (500 ng/ml) for an additional 24 h. Then, the SW480 cells were fixed, stained with PI, and subjected to flow cytometry analysis to probe the cell cycle. **(e-f)** SW480 and HCT116 cells were harvested and double stained with an FITC-conjugated anti-Annexin V antibody and PI. Then, the cells were subjected to flow cytometry for cell apoptosis analysis. **(g)** The protein levels of cyclin D1, B, E, Bcl-2, Bax, and cleaved caspase-3 in response to LRG1 treatment in SW480 cells were analysed by Western blot. This figure shows representative images of repeated experiments, and the data are presented as the mean±standard deviation. Compared with NC, *P<0.05, **P<0.01, and ***P<0.001.

References

1. IARC, Globocan 2012.World Health Organization.Paris.2014 Available from: URL: <http://globocan.iarc.fr/Default.aspx>.

2. Ludwig JA, Weinstein JN.Biomarkers in cancer staging, prognosis and treatment selection.Nature Reviews Cancer.2005. 5:845–56.

3. Duffy MJ, van Dalen A, Haglund C, Hansson L, Holinski-Feder E, Klapdor R, et al.Tumour markers in colorectal cancer: European Group on Tumour Markers (EGTM) guidelines for clinical use.European Journal of Cancer.2007. 43:1348–60.

4. MJ, Duffy.Carcinoembryonic antigen as a marker for colorectal cancer: Is it clinically useful?Clinical Chemistry.2001. 47:624–30.

5. Haupt H, Baudner S.Isolation and characterization of an unknown, leucinerich 3.1-S- alpha2-glycoprotein from human serum.Hoppe Seyler Z Physiol Chem.1977. 358(6):639-46.

6. Ladd JJ, Busald T, Johnson MM, Zhang Q, Pitteri SJ, Wang H, et al.Increased plasma levels of the apc-interacting protein mapre1, lrg1, and igfbp2 preceding a diagnosis of colorectal cancer in women.Cancer Prev Res (Phila).2012:655-64.

7. Kentsis A, Ahmed S, KurekK, Brennan E, Bradwin G, Steen H, et al.Serum leucine-rich alpha-2 glycoprotein is a disease activity biomarker in ulcerative colitis.Ann Emerg Med.2012. 60:78-83.

8. Serada S, Fujimoto M, Terabe F, Iijima H, Shinzaki S, Matsuzaki S, et al.Serum leucine-rich alpha-2 glycoprotein is a disease activity biomarker in ulcerative colitis.Inflamm Bowel Dis.2012. 18:2169–79.

9. Hütter G, Neumann M, Nowak D, Klein S, Klüter H, Hofmann W-K.The effect of the ccr5-delta32 deletion on global gene expression considering immune response and inflammation.J Inflamm.2011. 8:29.

10. Wang X, Abraham S, McKenzie JA, Jeffs N, Swire M, Tripathi VB, et al.LRG1 promotes angiogenesis by modulating endothelial TGF-β signalling.Nature.2013. 499:306–11.

11. He X, Wang Y, Zhang W, Li H, Luo R, Zhou Y, et al.Screening differential expression of serum proteins in afp-negative hbv-related hepatocellular carcinoma using iTRAQ–MALDI-MS/MS.Neoplasma.2013. 61:17-26.

12. Uen YH, Lin KY, Sun DP, Liao CC, Hsieh MS, Huang YK, et al.Comparative proteomics, network analysis and post-translational modification identification reveal differential profiles of plasma con a-bound glycoprotein biomarkers in gastric cancer.J Proteomics.2013. 83:197-213.

13. Furukawa K, Kawamoto K, Eguchi H, Tanemura M, Tanida T, Tomimaru Y, et al.Clinicopathological Significance of Leucine-Rich α2-Glycoprotein-1 in Sera of Patients With Pancreatic Cancer.Pancreas.2015. 44:93-8.

14. Wu RC, Yu CS, Liu KC, Huang HY, Ip SW, Lin JP, et al.Citosol (thiamylal sodium) triggers apoptosis and affects gene expressions of murine leukemia raw 264.7 cells.Hum Exp Toxicol.2012. 31:771-9.

15. Wu J, Xie X, Nie S, Buckanovich RJ, Lubman DM.Altered expression of sialylated glycoproteins in ovarian cancer sera using lectinbased elisa assay and quantitative glycoproteomics analysis.J Proteome Res.2013. 12:3342–52.

16. Lindén M, Lind SB, Mayrhofer C, Seqersten U, Wester K, Lyutvinskiy Y, et al.Proteomic analysis of urinary biomarker candidates for nonmuscle invasive bladder cancer.Proteomics.2012. 12:135-44.

17. Liu Y, Luo X, Hu H, Wang R, Sun Y, Zeng R, et al.Integrative proteomics and tissue microarray profiling indicate the association between overexpressed serum proteins and non-small cell lung cancer.PLoS One.2012. 7.

18. Anglin I, Passaniti A.Runx protein signaling in human cancers.Cancer Treat Res.2004. 119:189-215.

19. Coffman JA.Is Runx a linchpin for developmental signaling in metazoans?J Cell Biochem.2009. 107:194-202.

20. Kidwai F, Edwards J, Zou L, Kaufman DS.Fibrinogen Induces RUNX2 Activity and Osteogenic Development from Human Pluripotent Stem Cells.Stem Cells.2016. 22.

21. Bangsow C, Rubins N, Glusman G, Bernstein Y, Negreanu V, Goldenberg D, et al.The RUNX3 gene—sequence, structure and regulated expression.Gene.2001. 279:221-32.

22. Lee KS, Hong SH, Bae SC.Both the Smad and p38MAPK pathways play a crucial role in Runx2 expression following induction by transforming growth factorbeta and bone morphogenetic protein.Oncogene.2002. 21:7156-63.

23. Cameron ER, Blyth K, Hanlon L, Kilbey A, Mackay N, Stewart M, et al.The Runx genes as dominant oncogenes.Blood Cells Mol Dis.2003. 30:194-200.

24. Ito Y, Osato M, Ito K.RUNX and cancer.Ann Acad Med Singapore.2003. 32:S6-S7.

25. Zhang J, Zhu L, Fang J, Ge Z, Li X.LRG1 modulates epithelial-mesenchymal transition and angiogenesis in colorectal cancer via HIF-1α activation.J Exp Clin Cancer Res.2016. 35：29.

26. Choi JW, Liu H, Shin DH, Yu G, Hwang JS, Kim ES.Proteomic and cytokine plasma biomarkers for predicting progression from colorectal adenoma to carcinoma in human patients.Proteomics.2013. 13:2361–74.

27. Zhang Y, Luo Q, Wang N, Hu F, Jin H, Ge T, et al.LRG1 suppresses the migration and invasion of hepatocellular carcinoma cells.Med Oncol.2015. 32:146.

28. Wen SY, Zhang LN, Yang XM, Zhang YL, Ma L, Ge QL, et al.LRG1 is an independent prognostic factor for endometrial carcinoma.Tumour Biol.2014. 35(7):7125-33.

29. Wang X, Abraham S, McKenzie JA, Jeffs N, Swire M, Tripathi VB, et al.LRG1 promotes angiogenesis by modulating endothelial TGF-beta signalling.Nature.2013. 499(7458):306-11.

30. Zhong D, He G, Zhao S, Li J, Lang Y, Ye W, et al.LRG1 modulates invasion and migration of glioma cell lines through TGF-β signaling pathway.Acta Histochem.2015. 117:551-8.

31. Sherr CJ.Cancer cell cycles.Science.1996. 274:1672–7.

32. Hochegger H, Takeda S, Hunt T.Cyclin-dependent kinases and cellcycle transitions: does one fit all?Nat Rev Mol Cell Biol.2008. 2008(9):910-6.

33. Simpson DJ, Fryer AA, Grossman AB,Wass JA, Pfeifer M, Kros JM, et al.Cyclin d1 (ccnd1) genotype is associated with tumour grade in sporadic pituitary adenomas. Carcinogenesis.2001. 22:1801–7.

34. Zhong D, Zhao S, He G, Li J, Lang Y, Ye W, et al.Stable knockdown of LRG1 by RNA interference inhibits growth and promotes apoptosis of glioblastoma cells in vitro and in vivo.Tumor Biol.2015. 36(6):4271–8.

35. Lund AH, van Lohuizen M.RUNX: a trilogy of cancer genes.Cancer Cell.2002. 1(3):213-5.

36. Suárez-Villanueva S, Ayala-Madrigal ML, Peregrina-Sandoval J, Macías-Gómez N, Ramírez-Ramírez R, Muñiz-Mendoza R, et al.RUNX3 gene polymorphisms and haplotypes in Mexican patients with colorectal cancer.Genet Mol Res.2015. 14(4):15505-10.

37. Mu WP, Wang J, Niu Q, Shi N, Lian HF.Clinical significance and association of RUNX3 hypermethylation frequency with colorectal cancer: a meta-analysis.Onco Targets Ther.2014. 7:1237-45.

38. Ito Y, Miyazono K.RUNX transcription factors as key targets of TGF-beta superfamily signaling.Curr Opin Genet Dev.2003. 13(1):43-7.

39. Blyth K, Cameron ER, Neil JC.The RUNX genes: gain or loss of function in cancer.Nat Rev Cancer.2005. 5(5):376-87.

40. Slattery ML, Lundgreen A, Herrick JS, Caan BJ, Potter JD, Wolff RK.Associations between genetic variation in RUNX1, RUNX2, RUNX3, MAPK1 and eIF4E and riskof colon and rectal cancer: additional support for a TGF-β-signaling pathway.Carcinogenesis.2010. 32(3):318-26.

41. Liu HP, Cao AT, Feng T, Li Q, Zhang W, Yao S, et al.TGF-β converts Th1 cells into Th17 cells through stimulation of Runx1 expression.Eur J Immunol.2015. 45(4):1010-8.
